# The prevalence of *Toxoplasma gondii* in mice living in Danish indoor sow herds

**DOI:** 10.1186/s13028-019-0483-z

**Published:** 2019-10-16

**Authors:** Stine Thorsø Nielsen, Isabella Linde Westergaard, Grith Kirkhoff Guldbech, Henrik Vedel Nielsen, Maria Vang Johansen

**Affiliations:** 1 Section for Parasitology and Aquatic Pathobiology, Department of Veterinary and Animal Sciences, University of Copenhagen, Grønnegårdsvej 15, 1870 Frederiksberg, Denmark; 2 Statens Serum Institut, Artillerivej 5, 211/237, 2300 Copenhagen, Denmark

**Keywords:** Indoor sow herds, *Mus musculus*, *Sus scrofa domesticus*, Transmission, *Toxoplasma gondii*

## Abstract

**Background:**

*Toxoplasma gondii* is found worldwide, and consumption of undercooked meat is considered a significant risk factor for human infections. In Denmark, little is known about the distribution of *T. gondii*, but a recent study revealed a seroprevalence of 34% in Danish indoor sows. The present cross-sectional study aimed to investigate the role of mice for the transmission of *T. gondii* in Danish indoor sow herds.

**Results:**

In total, 56 sow herds were visited, 137 mice were caught by snap traps from 32 farms, and 52 cat faecal samples were collected from 22 farms. Eight percent of the mice were positive for *T. gondii* DNA, representing 11% of the farms. Significant associations were found between the presence of *T. gondii*-positive mice and both open feed systems (P= 0.041) and extra rodent control on the farm (P= 0.024). All cat faecal samples were deemed negative for *T. gondii* by light microscopy examination and real-time polymerase chain reaction analysis.

**Conclusion:**

Mice captured inside Danish sow herds were found to be infected with *T. gondii* and may thus contribute to the transmission of *T. gondii* to sows, which may explain the high seroprevalence found in Danish pigs.

## Background

*Toxoplasma gondii* is a zoonotic parasite found worldwide, and up to one-third of the human population is estimated to be infected [1]. *T. gondii* infected pork is considered as an important source of *T. gondii* infection for humans in Europe and USA [1, 2]. Apart from consuming raw or undercooked meat, humans may become infected from oocyst contaminated soil, vegetables and water, or directly from cats excreting oocysts in their faeces [3]. Infection with *T. gondii* is generally asymptomatic or cause mild symptoms only, but can cause severe disease in immunocompromised people and children infected prenatally [4]. Additionally, infection with *T. gondii* has been associated with the development of psychiatric disorders like schizophrenia [5, 6]. Preventive measures should allow for *T. gondii*-free animal productions when using intensive indoor housing systems for pigs as widely practiced in e.g. Denmark [1]. Rodent control has been found to significantly reduce the transmission of *T. gondii* to sows [7–10]. However, a recent study on Danish abattoirs measured a *T. gondii* seroprevalence of 33.7% in Danish indoor sows [11]. The present study aimed to investigate the potential role of mice for the transmission of *T. gondii* in Danish indoor sow herds by i) determining the prevalence of *T. gondii* in mice caught in sow herds, and ii) investigating if risk factors for porcine toxoplasmosis were present in Danish indoor sow herds. Additionally, the excretion of *T. gondii* oocysts from cats on farms having indoor sows was investigated.

## Methods

**Fig. 1:**
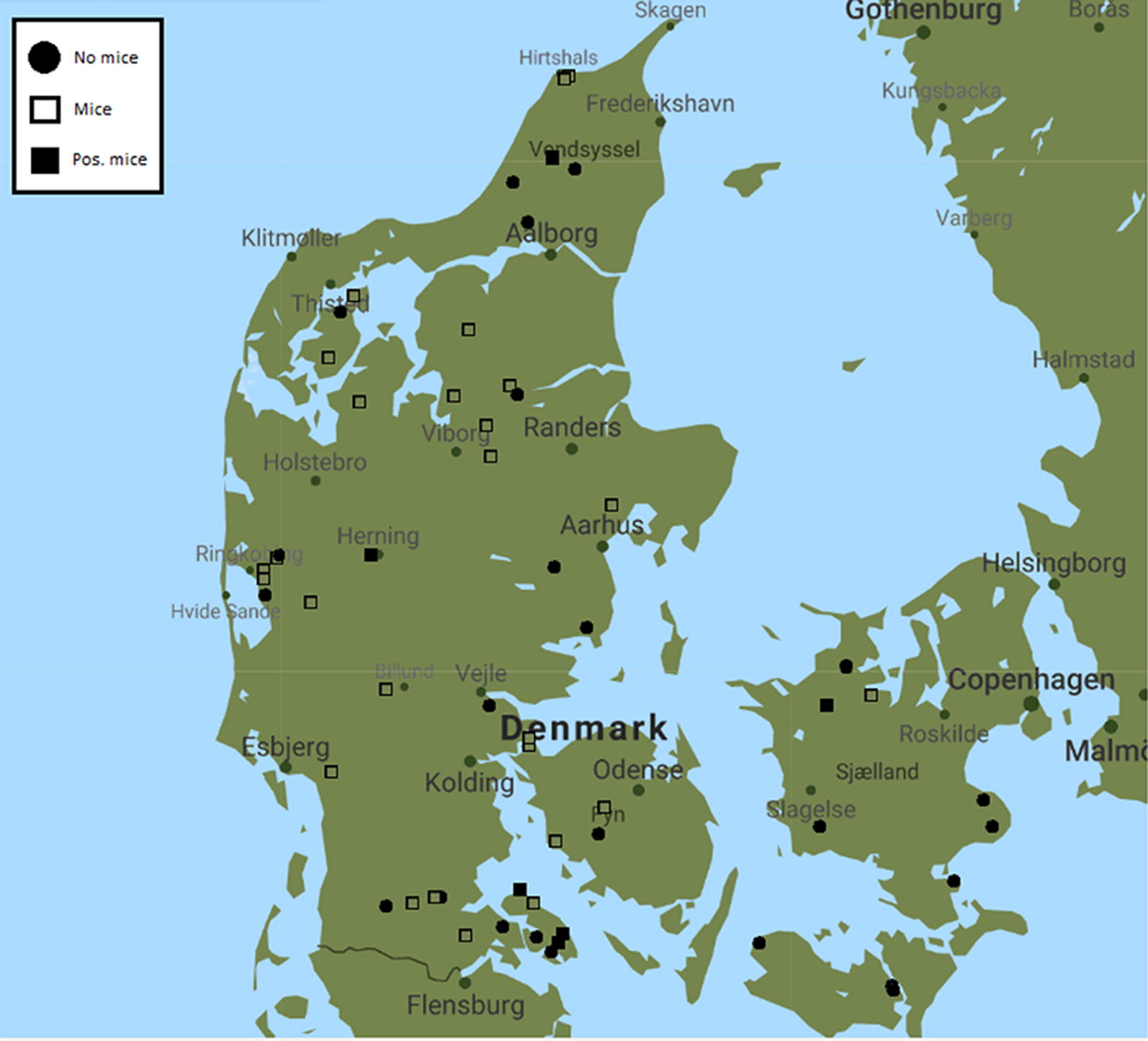
Map of Denmark showing the location of the farms and status of the mice caught on them

The study was a cross-sectional study, where the target sample size was calculated using an assumed prevalence of *T. gondii* in mice of 6.5% [12], an allowable error of 0.1 and a 95% confidence interval. Adjusted by the total numbers of sow herds in Denmark, N = 570 [13], the target sample size became 23 farms. Farms were randomly selected by a SAS 9.2 random number generator based on the criteria of (i) having a minimum of 200 sows in the herd, and (ii) being a breeding and multiplier herd, a production herd or a weaner multiplier herd. Farms located on the islands Bornholm, Langeland and Orø were excluded for logistical reasons. Listed farm owners were recruited by email or telephone. Data were collected from December 2017 to March 2018. The sow herds were dispersed across the country as shown in Fig. 1.

### Collection of data and samples

Data and sample collection comprised collection of mice and cat faeces, a questionnaire interview and an observational study. Each farm was visited once during the normal working hours for 2–4 h, and again the following day. The farm owner or the manager was interviewed during the first visit. In one case, the questionnaire was emailed and filled out by the manager. On each farm, 16 mouse snap traps were installed, of which eight traps were inside the pigsty and eight were outside the pigsty, i.e. in open storage spaces or along the outer walls of the pigsty. The traps were left overnight and collected the next day, using raisins and peanut butter as bait. In three cases, the traps were left for two nights, to comply with visitor quarantine rules. The mice were weighed, measured and characterised to determine the species. The brain from each mouse was sampled and immediately stored in a freezer box until return to the laboratory after which it was stored at − 20 °C until further analysis. From cats belonging to the farms and having indoor access, faecal samples were collected. In cases where the cats did not defecate during the visit, faecal samples were collected from the floor or from existing litter boxes. The questionnaire was designed to gather information about the daily routines on the farm, pig management, feed storage, biosecurity and presence and management of cats and mice. The questions were created partly based on the standardised online biosecurity questionnaire [14] (BioCheck.ugent^®^Pig, 2018). As BioCheck only covers general biosecurity issues on pig farms, questions regarding the specific transmission of *T. gondii* were included [11, 15, 16]. The observational survey was designed to describe the actual and current state of each farm in relation to management, housing, biosecurity, cat and mice abundance and their access to the pigsty and surroundings. An observer guide was developed, and recording took place during each visit. Prior to the farm visits, clear definitions of response options and observations were made. A closed feed system was defined as a system, where pig feed was stored in sealed silos and transported to the sows through pipes. A feed system was defined as open, if the pig feed was accessible for mice or cats at any time in the system, e.g. an open silo, open grain storage or a leakage.

### DNA extraction, microscopy and *T. gondii* identification with PCR

Using a QIAamp Mini Kit (QIAGEN: cat. no./ref. 51,306, Qiagen, Hilden, Germany), DNA was extracted from a subsample (approximately 25 mg) of each mouse brain. Cat faecal samples were examined on the day of collection. Using a McMaster technique [17], 4 g of faeces were examined in a special made McMaster chamber by a light microscope at 40× objective magnification for *T. gondii* oocysts using flotation fluid with MgSO_4_ (sg. 1.280) [18]. Subsequent sample preparation and analysis was performed at Statens Serum Institut, Copenhagen. From the cat faeces, DNA was extracted through a NucliSENS^®^ easyMAG^®^ (bioMérieux, France), using Protocol Specific B 2.0.1 as described by Mirsepasi et al. [19]. After extraction, 50 μL eluate was transferred to sterilised 1.5 mL Eppendorf tubes and run in a real-time polymerase chain reaction (PCR) analysis [20] with the 529 bp gene as a specific for *T. gondii* [21]. For the real-time PCR, the reaction volume was 50 μL including 5 μL purified DNA from either cat faeces or mouse brain.

### Statistical analysis

Descriptive statistics were applied at the farm level. Odds ratios (OR) were calculated and used to describe the strength of association between two variables. A significance level of 5% was used. Statistical analyses were performed in Microsoft Excel 2011/2013 and R version 3.5.0 [22].

## Results

A total of 56 farms were visited, and 137 mice were caught on 32 different farms. The proportion of mice caught indoor were 130, and seven mice were caught outdoor. The mice were identified as *Mus musculus* (n = 82), *Apodemus sylvaticus* (n = 27), *Apodemus flavicollis* (n = 5), and unidentified (n = 23) due to immaturity. No voles or shrews were caught. The prevalence of *T. gondii* in mice was 8% (11/137), and the prevalence of farms with positive mice was 11% (6/56). All positive mice were caught inside the pigsty and identified as *M. musculus*.

Based on the questionnaire survey, 49 farmers stated that mice were abundant on their farms and could gain access to indoor pigsty areas, and 21 farmers stated to perform extra rodent control as installing snap traps or using poison, of which 12 used rat poison. Mice or traces after mice were observed inside the pigsty on 26 farms. On five farms, it was observed that mice lived in transponder feed stations, and it was observed on four farms that mice lived in the deep litter among the pigs. Mice had access to pig feed via open feed systems on 15 farms. The feed grinder was accessible to mice on eight farms. Significant associations are shown in Table 1.

**Table 1:** Positive mice tested pairwise for conditional independence (OR = 1) with corresponding odds ratio (OR), 95% CI and P value

Outcome variable	Exposure variable	Odds ratio	95% CI	P value
Positive mice	Extra rodent control	10.17	(1.02, 515.31)	*0.024*
	Open feed system	6.61	(0.82, 82.04)	*0.041*
	Feed residues	0.48	(0.06, 3.97)	0.397
	Deep litter bedding	0.97	(0.08, 7.59)	1

Fifty-two cat faecal samples were collected from 22 different farms. None of the samples were positive for *T. gondii* oocysts by light microscope analysis or real-time PCR.

## Discussion

Mice had access to feeding and grinding systems and to the pig pens, which suggest that they represent a significant risk for transmitting *T. gondii* to the sows. The access of rodents to feed stations has been found to increase the risk of *T. gondii* transmission significantly [9]. It is assumed that feed residues attracts mice, which would be possible in open feed systems, feed grinders and transponder feed stations.

The results showed that the odds of catching mice and *T. gondii*-positive mice were significantly higher on farms where extra rodent control was performed. Previous results report a decreased prevalence of toxoplasmosis in the pigs when using rodent control [7, 9, 10, 23]. This suggests that the extra rodent control measures are insufficient, or that farmers experiencing rodent problems may be more prone to use extra rodent control measures. In Europe, the use of rat poison with anticoagulants has been restricted but is still used in cases with evidence or strong indications of rats present on the farm. It is possible for farmers in Denmark to get a certification that enables them to use the poison on their farm. Anticoagulants cause the mice to die from internal bleedings, and if the mice end up dying in the pig pens, sows have been observed to eat the mice. Similarly, disposing dead mice into the pens or leaving them on the floor may increase the risk of the sows eating potentially infected mice (Hansen SV, University of Copenhagen, personal communication).

To our knowledge, no study has investigated the association between deep litter bedding and mice abundance. Mice are assumed to live in the deep litter bedding, and due to the rare cleaning of it, they can stay here for a long period of time. This study did not find any significant association between the presence of mice and farms with deep litter bedding in the pens. The role of deep litter bedding for the transmission of *T. gondii* to the pigs should be further investigated. Moreover, the location of the tissue cysts in the brain could be of importance. According to Vyas et al. [24], *T. gondii* tissue cysts are often located in the amygdala in the brain of mice. The extent of decay of the mouse brain tissue varied in the brain samples, which made identification of the amygdala difficult and could have led to an underestimation of the true prevalence. Additionally, the sensitivity would have been higher if the DNA extraction was done according to Opsteegh et al. [25] with magnetic capture prior to the PCR.

None of the 52 cat faecal samples proved positive for *T.* gondii oocysts, and the presence of cats on the farms did not increase the odds of *T. gondii* infection. Cats are generally believed to shortly excrete oocysts during a primary infection [26], and studies investigating oocyst excretion in cats have found very low prevalence of 0.31% [27] and 0.76% [28]. Thus, the likelihood of sampling faeces from an oocyst-excreting cat in this study was low, given the small sample size of cats. To determine the prevalence in cats, the sample size should be increased. Alternatively, the seroprevalence in Danish cats should be investigated, as an indicator for their exposure to *T. gondii* and their potential role in the epidemiology of this parasite on farms.

For future studies of this kind, tissue samples from both mice and swine should be collected, and PCR-positive tissues should be characterized by molecular genotyping technique, as done by Jokelainen et al. [20] to determine if mice in fact is a source of transmission of *T. gondii* to sows. Unfortunately, tissue sampling of the sows was not possible, and the farmers were volunteering despite of their high precaution in allowing people into the pigsty. However, this might be possible if the sows were followed from farm to slaughterhouse.

## Conclusions

Mice captured inside Danish sow herds were found to be infected with *T. gondii* and may thus contribute to the transmission of *T. gondii* to sows, which may explain the high prevalence found among Danish pigs. Further studies are warranted to fully elucidate the transmission of *T. gondii* in Danish indoor sow herds.

## Data Availability

The datasets used and analysed during the current study are available from the corresponding author on reasonable request.

## Abbreviations

PCR: polymerase chain reaction
CI: confidence interval
OR: odds ratio

## References

[1] Tenter A, Heckeroth AR, Weiss LM (2000). *Toxoplasma gondii*: from animals to humans. Int J Parasitol.

[2] Guo M, Dubey JP, Hill D, Buchanan RL, Gamble HR, Jones JL, Pradhan AK (2015). Prevalence and risk factors for *Toxoplasma gondii* infection in meat animals and meat products destined for human consumption. J Food Prot.

[3] Dubey JP (2004). Toxoplasmosis—a waterborne zoonosis. Vet Parasitol.

[4] Hill D, Dubey JP (2002). *Toxoplasma gondii*: transmission, diagnosis, and prevention. Clin Microbiol Infect.

[5] Burgdorf KS, Trabjerg BB, Pedersen MG, Nissen J, Banasik K, Pedersen OB (2019). Large-scale study of *Toxoplasma* and Cytomegalovirus shows an association between infection and serious psychiatric disorders. Brain Behav Immun.

[6] Webster JP, Kaushik M, Bristow GC, McConkey GA (2013). Toxoplasma gondii infection, from predation to schizophrenia: can animal behaviour help us understand human behaviour. J Exp Biol.

[7] Assadi-Rad AM, New JC, Patton S (1995). Risk factors associated with transmission of *Toxoplasma gondii* to sows kept in different management systems in Tennessee. Vet Parasitol.

[8] García-Bocanegra I, Simon-Grifé M, Dubey JP, Casal J, Martín GE, Cabezón O (2010). Seroprevalence and risk factors associated with *Toxoplasma gondii* in domestic pigs from Spain. Parasitol Int.

[9] Piassa FR, de Araujo JB, da Rosa RC, Mattei RJ, da Silva RC, Langoni H (2010). Prevalence and risk factors for *Toxoplasma gondii* infection in certified and non-certified pig breeding farms in the Toledo microregion, PR Brazil. Rev Bras Parasitol Vet..

[10] Herrero L, Gracia MJ, Pérez-Arquillué C, Lázaro R, Herrera M, Herrera A (2016). *Toxoplasma gondii*: pig seroprevalence, associated risk factors and viability in fresh pork meat. Vet Parasitol.

[11] Kofoed KG, Vorslund-Kiær M, Nielsen HV, Alban L, Johansen MV (2017). Seroprevalence of *Toxoplasma gondii* in Danish pigs. Vet Parasitol: Reg Stud Reports..

[12] Kijlstra A, Meerburg B, Cornelissen J, De Craeye S, Vereijken P, Jongert E (2008). The role of rodents and shrews in the transmission of *Toxoplasma gondii* to pigs. Vet Parasitol.

[13] Helverskov O. Country average for pig production productivity 2016 [*Landsgennemsnit for produktivitet i svineproduktion 2016*]. SEGES Svineproduktion. 2017. Notat_1716. Accessed 25 Sep 2019.

[14] BioCheck.ugent^®^Pig. Biocheck.ugent. https://www.biocheck.ugent.be/ (2018). Accessed 10 Jul 2018.

[15] Lundén A, Lind P, Engvall EO, Gustavsson K, Uggla A, Vågsholm I (2002). Serological survey of *Toxoplasma gondii* infection in pigs slaughtered in Sweden. Scand J Infect Dis.

[16] Limon G, Beauvais W, Dadios N, Villena I, Cockle C, Blaga R (2017). Cross-sectional study of *Toxoplasma gondii* infection in pig farms in England. Foodborne Pathog Dis..

[17] Roepstorff A, Nansen P. *FAO Animal Health Manual No. 3* - *Epidemiology, diagnosis and control of helminth parasites of swine*. Food and Agriculture organization of the United Nations. 1998. http://www.fao.org/3/a-x0520e.pdf. Accessed 10 Dec 2017.

[18] Cringoli G. *FLOTAC manual appendix No. 1* - *Herbivores: Flotation solutions and parasitic elements.* 1st ed. Veterinary Parasitology and Parasitic Diseases, Department of Pathology and Animal Health, Faculty of Veterinary Medicine, University of Naples Federico II; 2009.

[19] Mirsepasi H, Persson S, Struve C, Andersen LOB, Petersen AM, Krogfelt KA (2014). Microbial diversity in fecal samples depends on DNA extraction method: easyMag DNA extraction compared to QIAamp DNA stool mini kit extraction. BMC Res Notes..

[20] Jokelainen P, Murat JB, Nielsen HV (2018). Direct genetic characterization of *Toxoplasma gondii* from clinical samples from Denmark: not only genotypes II and III. Eur J Clin Microbiol Infect Dis.

[21] Homan WL, Vercammen M, De Braekleer J (2000). Identification of a 200- to 300-fold repetitive 529 bp DNA fragment in *Toxoplasma gondii*, and its use for diagnostic and quantitative PCRp. Int J Parasitol.

[22] R Core Team. R: A Language and Environment for Statistical Computing, 2018.

[23] García-Bocanegra I, Dubey JP, Simon-Grifé M, Cabezón O, Casal J, Allepuz A (2010). Seroprevalence and risk factors associated with *Toxoplasma gondii* infection in pig farms from Catalonia, north-eastern Spain. Res Vet Sci.

[24] Vyas A, Kim SK, Giacomini N, Boothroyd JC, Sapolsky RM (2007). Behavioral changes induced by *Toxoplasma* infection of rodents are highly specific to aversion of cat odors. Proc Natl Acad Sci.

[25] Opsteegh M, Langelaar M, Sprong H, den Hartog L, De Craeye S, Ajzenberg D (2010). Direct detection and genotyping of *Toxoplasma gondii* in meat samples using magnetic capture and PCR. Int J Food Microbiol.

[26] Dubey JP (2010). Toxoplasmosis of animals and humans.

[27] Schares G, Globokar Vrhovec M, Pantchev N, Herrmann DC, Conraths FJ (2008). Occurrence of *Toxoplasma gondii* and *Hammondia hammondi* oocysts in the faeces of cats from Germany and other European countries. Vet Parasitol.

[28] Jokelainen P, Simola O, Rantanen E, Näreaho A, Lohi H, Sukura A (2012). Feline toxoplasmosis in Finland: cross-sectional epidemiological study and case series study. J Vet Diagn Invest.

